# A combined valve-sparing aortic root replacement and primary tetralogy of Fallot repair in an unoperated adult: a case report

**DOI:** 10.3389/fcvm.2026.1851997

**Published:** 2026-08-24

**Authors:** Xuping Feng, Dou Yuan, Juan Wu, Yongjun Qian

**Affiliations:** 1 Anesthesia Operation Center of West China Hospital, Sichuan University, Chengdu, Sichuan, China; 2 Department of Cardiovascular Surgery, Chengdu Shang Jin Nan Fu Hospital, West China Hospital of Sichuan University, Chengdu, Sichuan, China; 3 Out-patient Department, West China Hospital, Sichuan University, Chengdu, Sichuan, China; 4 Department of Cardiovascular Surgery, West China Hospital, Sichuan University, Chengdu, Sichuan, China

**Keywords:** aortic root aneurysm, congenital heart disease, tetralogy of Fallot, valve-sparing aortic root replacement, Yacoub remodeling technique

## Abstract

**Background:**

Progressive aortic root dilation represents a life-threatening sequela of uncorrected tetralogy of Fallot (TOF), predisposing patients to aortic regurgitation, dissection, and rupture. Valve-sparing aortic root replacement (VSARR) offers an attractive alternative to mechanical valve replacement by eliminating prosthesis-related complications and lifelong anticoagulation. However, experience with VSARR in unoperated adult TOF patients remains exceptionally limited.

**Case presentation:**

We report a 35-year-old male with unrepaired TOF who presented with progressive exertional dyspnea, cyanosis, and atrial fibrillation. Comprehensive imaging revealed severe aortic root dilation (55.4 mm), moderate-to-severe aortic regurgitation, perimembranous ventricular septal defect, infundibular stenosis, and secundum atrial septal defect. We performed single-stage surgical correction comprising ventricular septal defect closure, infundibular resection, pulmonary valvotomy with transannular patch, atrial septal defect closure with fenestration, and Yacoub remodeling VSARR. The patient tolerated the 208-min cardiopulmonary bypass procedure well, with immediate postoperative echocardiography demonstrating trivial aortic regurgitation. He was discharged on postoperative day 7 in sinus rhythm without complications.

**Conclusions:**

This case demonstrates the feasibility of combining Yacoub VSARR with primary intracardiac TOF repair in unoperated adults. The procedure successfully preserved native aortic valve function while correcting congenital anatomy, avoiding prosthetic valve-related morbidity. This approach may expand surgical options for adults with TOF-associated aortopathy, though long-term durability data are needed.

## Introduction

Tetralogy of Fallot (TOF) represents the most common cyanotic congenital heart defect, with surgical correction typically performed in infancy. However, in regions with limited healthcare access, a subset of patients reach adulthood without prior intervention. These individuals face a distinctive natural history characterized by chronic right ventricular pressure overload, progressive hypoxemia, and—critically—progressive dilation of the aortic root (1, 2).

The mechanism of aortic pathology in TOF is multifactorial. The overriding aorta receives both systemic and right ventricular output, subjecting it to chronic volume overload (2, 4). Additionally, abnormal flow patterns and intrinsic aortic wall abnormalities contribute to cystic medial degeneration (2). Over decades, this process produces aneurysmal dilation, aortic regurgitation, and heightened risk of dissection or rupture—catastrophic events carrying mortality approaching 100% without emergent intervention (1).

Conventional management of aortic root pathology has relied upon composite valve-graft replacement with mechanical prostheses. While durable, this approach mandates lifelong anticoagulation with warfarin, introducing risks of hemorrhage, thromboembolism, and lifestyle restrictions that prove particularly burdensome for young adults already managing repaired congenital heart disease (3).

Valve-sparing aortic root replacement (VSARR), pioneered by Sir Magdi Yacoub, offers an elegant physiologic solution. By resecting dilated sinuses and re-suspending the native valve within a tailored Dacron graft, VSARR restores root geometry, eliminates regurgitation, and removes dissection risk while preserving native valve tissue (3). This avoids both prosthetic valves and anticoagulation, maintaining normal hemodynamics and unrestricted lifestyle. Contemporary series demonstrate 85%–95% freedom from reoperation at 10 years (3, 5).

Despite these advantages, VSARR experience in congenital heart disease remains confined predominantly to patients with previously repaired defects and late-onset aortic pathology (3, 5). Recently, Nguyen et al. reported concomitant VSARR and intracardiac repair in a 30-year-old patient with unrepaired TOF and prior Blalock-Taussig shunt (6), and Adachi et al. described VSARR with complete TOF repair in a 27-year-old following prior palliative shunt (7). Nevertheless, experience in completely unoperated adults—without any prior cardiac intervention—remains extraordinarily scarce, and the optimal technical strategy for combined repair in this population has not been defined. We present this case to contribute detailed technical description of the Yacoub remodeling approach, emphasize specific perioperative management strategies, and highlight practical lessons applicable to similar patients.

## Case presentation

### Clinical history

A 35-year-old male presented with progressive exertional limitations. First diagnosed with TOF at age 5 years, he received no surgical or transcatheter intervention—reasons undocumented and now unrecallable. For three decades, he adapted to chronic cyanosis, developing progressive dyspnea on exertion requiring recovery after climbing two flights of stairs. Family members noted perioral cyanosis with exertion. He denied chest pain, syncope, squatting behavior, or systemic venous congestion symptoms. Medical history included successfully treated pulmonary tuberculosis at age 25. An incidental hepatic hemangioma detected on recent abdominal ultrasound prompted referral for comprehensive cardiac evaluation.

### Physical examination

The patient appeared chronically ill with central cyanosis. Vital signs revealed room-air oxygen saturation 84%, blood pressure 118/60 mmHg, and irregularly irregular pulse at 96 beats/minute. Jugular venous pressure was elevated to 7 cm. A hyperdynamic left parasternal heave was palpable. Cardiac auscultation revealed atrial fibrillation, a harsh 3/6 systolic ejection murmur radiating to the suprasternal notch, and a separate 4/6 pansystolic blowing murmur at the left lower sternal border. The pulmonary component of the second heart sound was absent. No diastolic murmurs were appreciated.

### Diagnostic imaging

**Figure 1:**
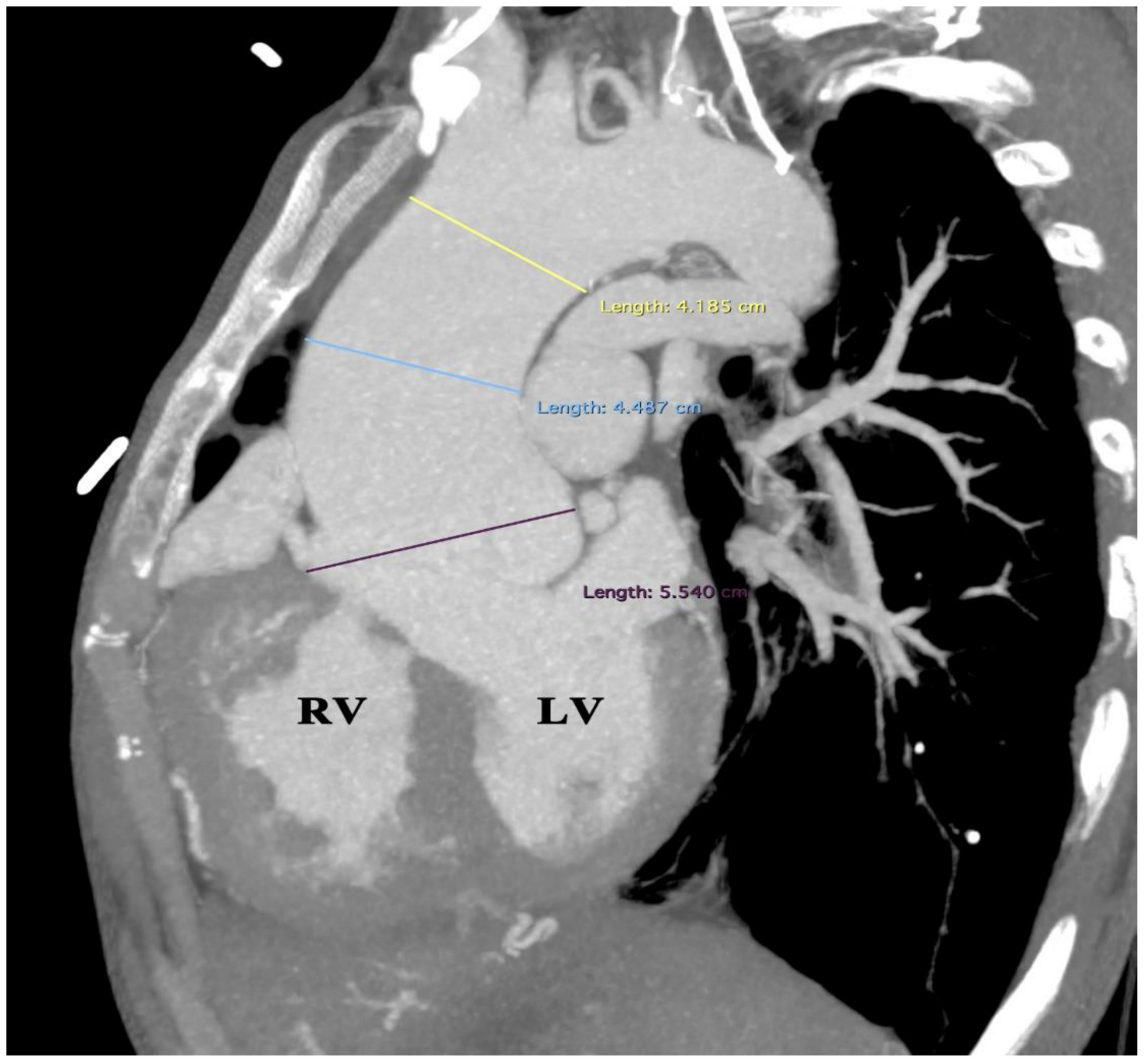
Preoperative computed tomographic angiography demonstrating aortic root dilation (55.4 mm) in the setting of unrepaired tetralogy of Fallot.

**Figure 2:**
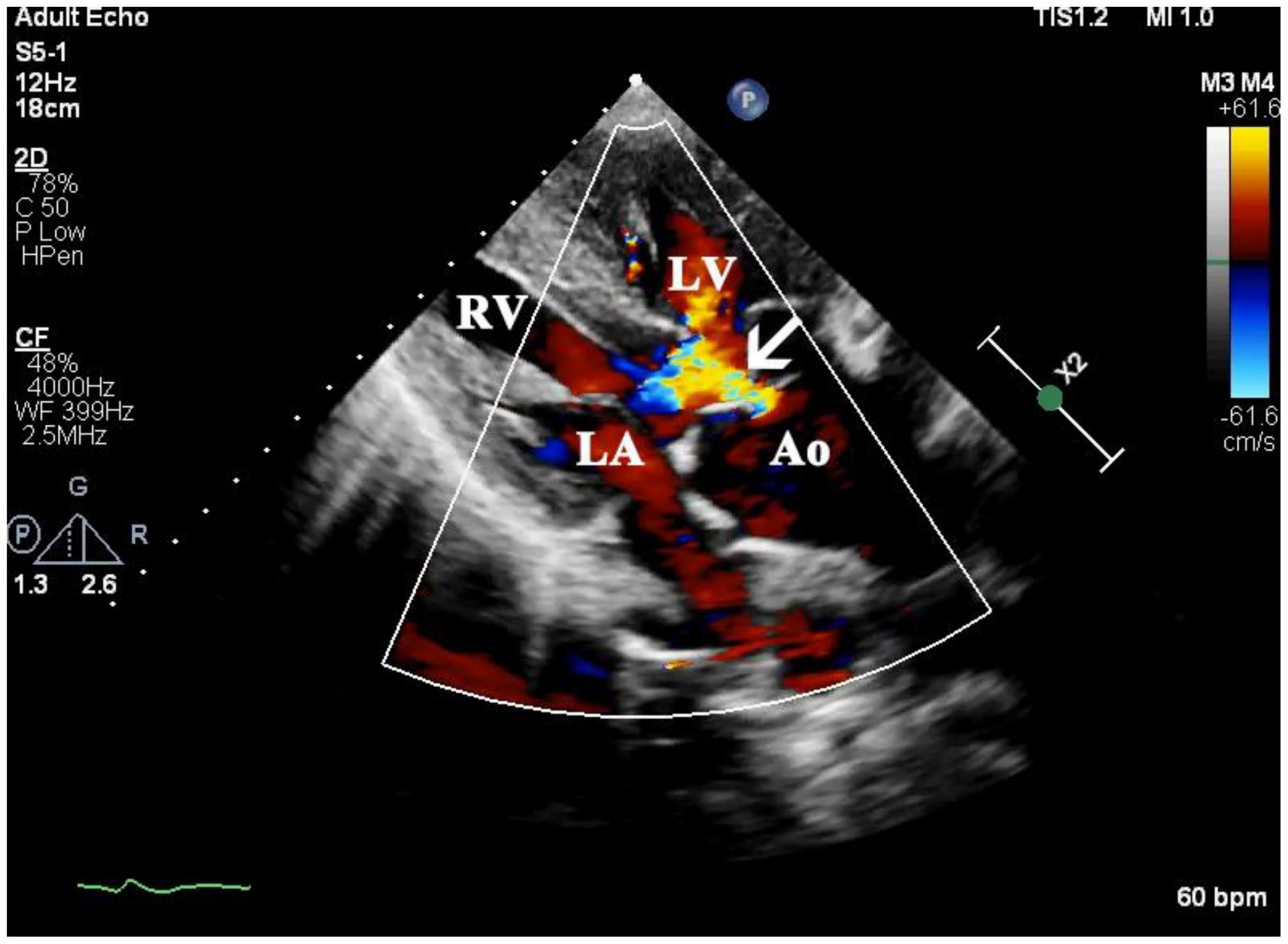
Preoperative transesophageal echocardiography showing dilated aortic root with color Doppler evidence of aortic regurgitation.

Transthoracic Echocardiography confirmed unrepaired TOF with severe secondary changes:

- Massively dilated right atrium (52 mm transverse diameter)
- Right ventricular hypertrophy (14 mm) with volume overload
- Severe sub pulmonary infundibular stenosis (minimal diameter 8 mm)
- Hypoplastic pulmonary annulus (18 mm) with doming leaflets; peak instantaneous gradient 64 mmHg
- Normal left ventricular size with preserved ejection fraction (73%)
- Aortic root overriding the septum by 50% with aneurysmal dilation (55.4 mm at sinuses of Valsalva) (1, 2) (Figure 1)
- Moderate-to-severe aortic regurgitation (vena contracta 5.7 mm)
- Dilated right coronary artery (7.5 mm) (Figure 2) (2)
- Moderate secundum atrial septal defect (16 mm) with bidirectional shunt
- Restrictive perimembranous ventricular septal defect (15 mm)

Computed Tomographic Angiography corroborated aortic root dilation and excluded coronary artery aneurysm.

### Multidisciplinary decision

Given the unique presentation of unoperated adult TOF with severe aortic root pathology, the congenital heart team recommended single-stage surgical correction. The proposed procedure integrated complete TOF repair with valve-sparing root replacement, offering simultaneous correction of intracardiac anatomy and aortic pathology while avoiding prosthetic valve-related complications (3, 7).

## Surgical procedure

### Cardiopulmonary bypass establishment

Through median sternotomy, aorto-bicaval cardiopulmonary bypass was established with a 22-Fr arterial cannula in the distal ascending aorta and two-stage venous cannulation. Systemic cooling to 32 °C was initiated. Myocardial protection employed antegrade cold blood cardioplegia (300 mL/min for 4 min) supplemented with intermittent retrograde cardioplegia every 20 min. A left atrial vent maintained a bloodless operative field.

### Intracardiac repair

**Figure 3:**
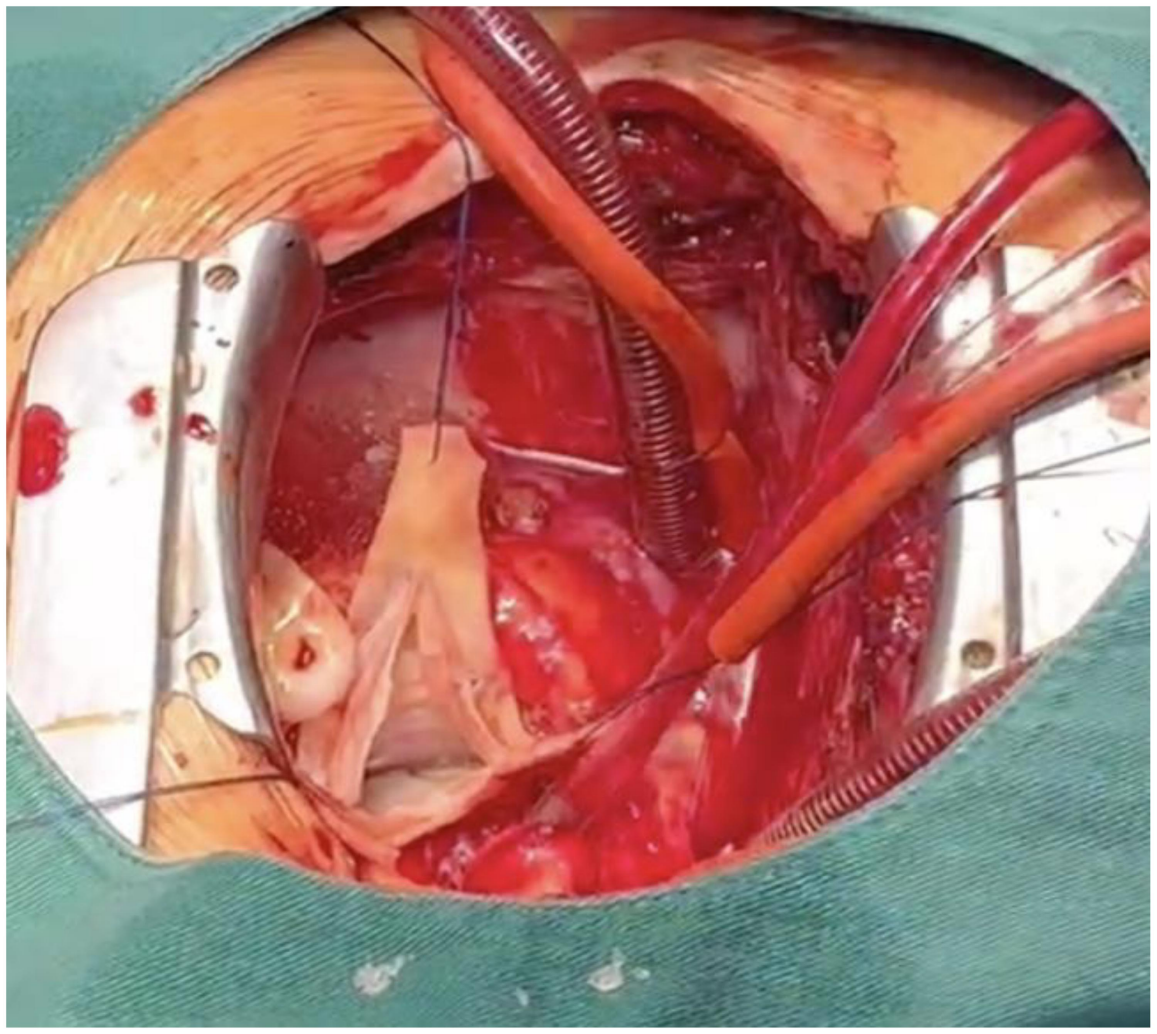
Intraoperative photograph revealing dilated aortic root with structurally normal aortic valve leaflets prior to valve-sparing procedure.

The right ventricle was opened via longitudinal infundibulotomy. Dense parietal and septal trabeculations were resected to expose the ventricular septal crest. A perimembranous inlet-outlet ventricular septal defect measured 15 × 12 mm, extending superiorly into the infundibulum. A 3 × 4 cm autologous pericardial patch, treated with 0.6% glutaraldehyde for 30 s, was secured with continuous 5-0 Prolene, employing shallow bites to avoid the conduction system (Figure 3).

### Valve-sparing aortic root replacement

The ascending aorta was transected 1 cm above the sinotubular junction. Inspection revealed thin, pliable trileaflet valves with effective height of 9 mm and no calcification or nodular thickening. All three sinuses were excised, preserving 5-mm arterial cuffs at the annulus. Both coronary ostia were mobilized on 8-mm buttons with circumferential epicardial dissection to ensure tension-free reimplantation (3).

A 26-mm woven Dacron graft was fashioned into a valvuloplasty ring: the proximal 3 mm was invaginated, doubled, and secured with running 4-0 Prolene to create a semi-rigid collar reproducing the sinotubular ridge. The native valve was seated within this collar, with commissures suspended by figure-of-eight 4-0 Prolene sutures at each commissural post apex. The proximal anastomosis was completed with continuous 4-0 Prolene immediately below each cusp nadir, creating three neosinuses. Coronary buttons were anastomosed to 8-mm aortic punch-created apertures using 5-0 Prolene with the parachute technique. The distal anastomosis was completed end-to-end with 4-0 Prolene (3).

### Pulmonary and atrial procedures

The 16-mm pulmonary trunk was enlarged with an autologous pericardial patch extending from annulus to bifurcation, achieving 22-mm diameter. The secundum atrial septal defect was partially closed, leaving a calibrated 3-mm fenestration as a right ventricular “pop-off” given anticipated compliance abnormalities.

### Weaning and intraoperative assessment

**Figure 4:**
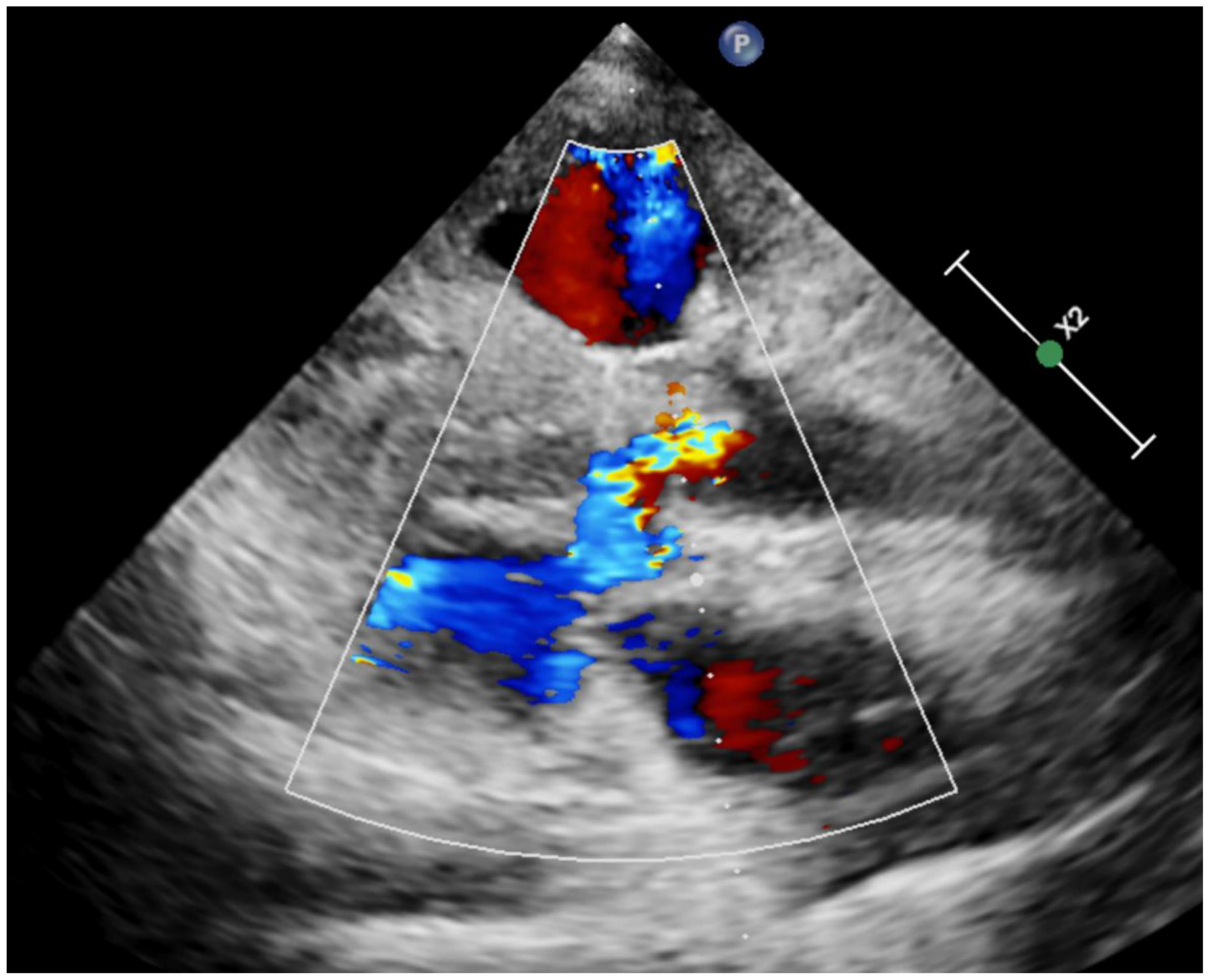
Postoperative color Doppler echocardiography demonstrating trivial residual aortic regurgitation following Yacoub remodeling valve-sparing aortic root replacement.

Rewarming to 36.5 °C preceded left heart de-airing and uneventful weaning from bypass without inotropic support. Transesophageal echocardiography (Figure 4) demonstrated:

- Trivial central aortic regurgitation (vena contracta 1.2 mm)
- Absent systolic anterior motion of the mitral valve
- Laminar coronary flow
- Direct right ventricular pressure measurement: 45 mmHg
- Pulmonary artery pressure: 18/8 mmHg

The chest was closed after meticulous hemostasis.

### Postoperative course and follow-up

The patient received a standardized anti-inflammatory protocol (methylprednisolone 15 mg/kg at induction, tight glycemic control <8 mmol/L, leukocyte-filtered bypass circuit) and oral amiodarone for 4 weeks (8–13). Despite prolonged cardiopulmonary bypass (208 min) and aortic cross-clamp (159 min), he remained in sinus rhythm throughout recovery without biochemical evidence of hepatic or renal injury. He was discharged on postoperative day 7.

## Discussion

### Comparison with existing literature

Concomitant VSARR and TOF repair in adults has been reported in only a handful of cases, making each contribution valuable for refining patient selection and technique. Adachi et al. described a 27-year-old with previously repaired TOF and prior Blalock-Taussig shunt who underwent David-V reimplantation with complete intracardiac repair (7). More recently, Nguyen et al. reported a 30-year-old with unrepaired TOF and prior BT shunt who underwent successful David procedure; at 6-month follow-up, the patient demonstrated trivial residual aortic regurgitation and improved left ventricular ejection fraction (from 42% to 55%) (6).

The present case differs in several clinically meaningful respects. First, our patient had no prior cardiac intervention—neither complete repair nor palliative shunt—resulting in more severe chronic cyanosis, right ventricular remodeling, and atrial fibrillation at presentation. Second, we employed the Yacoub remodeling technique rather than reimplantation, reflecting preserved sinus architecture and a desire to maintain physiologic root expansion. Third, the prolonged bypass time (208 min) in this completely unoperated adult necessitated specific anti-inflammatory and antiarrhythmic strategies that were not emphasized in prior reports. These distinctions highlight that VSARR can be integrated into diverse TOF anatomical and physiological contexts, but technique selection and perioperative management must be individualized.

### Pathophysiologic rationale

Progressive aortic dilation in TOF results from chronic exposure to combined pressure and volume overload. Right ventricular outflow obstruction generates retrograde systolic pressure transmission through the overriding aorta, while the right-to-left ventricular septal defect shunt imposes additional volume load (2, 4). Decades of stress induce cystic medial necrosis, elastic lamina fragmentation, and smooth muscle apoptosis—histologic changes mirroring those in Marfan syndrome despite differing etiology (2, 4).

### Valve-sparing strategy selection

Two principal VSARR techniques exist: the remodeling technique (Yacoub) and the reimplantation technique (David). The modified David-V operation, which recreates sinuses within a tubular graft while stabilizing the annulus, theoretically offers superior annular fixation and may be preferable when annular dilation is present or when the sinuses are severely attenuated (3). However, in this case, the Yacoub technique was selected because: (1) the native aortic sinuses remained remarkably preserved despite 35 years of volume overload, with thin, pliable leaflets and effective height of 9 mm; (2) physiologic root expansion characteristics were desired to maintain coronary flow dynamics; (3) the trileaflet valve demonstrated excellent mobility and geometric symmetry without annular dilation; and (4) the absence of connective tissue disorder made progressive annular dilation less likely (3). The immediate result—trivial central regurgitation—validates this strategy, though the reimplantation technique may offer superior long-term annular stability if root dilation recurs (3).

### Feasibility of valve-sparing root replacement in redo tetralogy of Fallot

An important question raised by this case is whether valve-sparing aortic root replacement is feasible in the more frequently encountered grown-up congenital heart disease (GUCH) scenario of previously repaired TOF complicated by late dilatation of the aortic root or ascending aorta. Late aortopathy is well documented in this population: progressive aortic root dilatation occurs in adults despite successful intracardiac repair (14), and in a multicenter cross-sectional study of 556 adults with repaired TOF, aortic root dimensions exceeding 40 mm were observed in approximately 30% of patients, with moderate or greater aortic regurgitation in 3.5% (15). When surgical intervention becomes indicated for rapid dimensional growth, an absolute diameter approaching 55 mm, or significant associated aortic regurgitation, the same valve-preserving principles described in this report can, in our opinion, be applied.

We believe that VSARR is feasible in carefully selected redo patients, provided that the native cusps remain structurally normal and pliable. Published experience supports this position: Baliulis et al. reported successful VSARR in adults previously operated for congenital heart defects, with excellent early valve competence (5), and the large pediatric series of Fraser and colleagues included reoperative cases without excess morbidity (3). Nevertheless, the redo setting introduces specific technical considerations. First, repeat sternotomy risks injury to the right ventricular outflow tract, transannular patch, or an aorta adherent to the posterior sternal table; preoperative computed tomographic assessment of retrosternal anatomy and, when necessary, peripheral cannulation prior to sternal entry are advisable. Second, adhesiolysis around the aortic root and coronary ostia must be meticulous to permit safe mobilization of coronary buttons and tension-free reimplantation. Third, because the aortopathy of repaired TOF reflects intrinsic medial degeneration that may progress after the index repair (14), we would favor the reimplantation (David) technique over remodeling in redo patients with annular dilatation or more than mild aortic regurgitation, given its superior annular stabilization; the remodeling approach remains appropriate when the annulus is of normal dimension and sinus geometry is preserved (3, 5). Finally, severely diseased, calcified, fenestrated, or prolapsing cusps constitute a contraindication to valve preservation, in which case composite root replacement remains the appropriate alternative. In summary, the feasibility of VSARR in redo TOF is determined primarily by cusp quality and annular dimensions rather than by reoperative status itself, and we anticipate that valve-sparing reoperations will assume an expanding role in the surgical management of TOF-associated aortopathy as surveillance intensifies.

### Rationale for atrial septal defect fenestration

The decision to leave a 3-mm fenestration in the secundum atrial septal defect was deliberate. Adult TOF patients exhibit chronic right ventricular hypertrophy with reduced compliance; sudden elimination of the right-to-left shunt after complete intracardiac repair can precipitate right ventricular failure and low cardiac output syndrome, particularly after prolonged bypass. The calibrated fenestration serves as a pressure pop-off, allowing transient right-to-left shunting until right ventricular compliance improves. This strategy is analogous to the fenestrated Fontan circulation and reduces perioperative morbidity in adult congenital repairs when right ventricular compliance is uncertain. We elected a 3-mm diameter based on estimated right ventricular end-diastolic pressure and anticipated postoperative compliance recovery.

### Inflammatory modulation in complex congenital surgery

The decision to combine procedures necessarily prolonged bypass and cross-clamp times, amplifying systemic inflammatory response. Experimental data demonstrate that cardiopulmonary bypass ≥120 min triggers 5- to 8-fold surges in IL-6, TNF-α, and complement C5a, correlating with endothelial dysfunction, capillary leak, and low cardiac output syndrome (8, 9). In adult congenital surgery, elevated IL-6 predicts prolonged vasopressor requirements and extended ICU stay (11).

Critically, this inflammatory milieu lowers atrial fibrillation threshold. Each additional 30 min of bypass increases postoperative AF odds by 26%, an effect mediated through inflammatory cytokines (12). Our anti-inflammatory bundle and amiodarone prophylaxis maintained sinus rhythm despite exceeding established risk thresholds, suggesting that inflammatory modulation may mitigate arrhythmic risk in complex congenital procedures (8–13).

### Technical considerations

Successful VSARR in this setting required meticulous attention to:

- Ventricular septal defect debridement without traction on the right coronary leaflet, given the absence of a subannular muscular rim characteristic of TOF (7)
- Patch sizing to avoid annular distortion, particularly because the VSD patch edge forms part of the subaortic annulus
- Coronary mobilization with generous buttons and complete epicardial dissection to prevent kinking after root remodeling (3)
- Graft sizing appropriate for the patient’s native annulus and cusp geometry, accounting for the horizontal aortic position typical in TOF
- Commissural suspension height to ensure adequate coaptation without systolic anterior motion

### Follow-up and surveillance limitations

The present report is limited to immediate postoperative findings through hospital discharge. Longer-term follow-up data, including serial echocardiographic assessment of aortic regurgitation severity, aortic root dimensions, right ventricular pressure and function, pulmonary valve competence, and rhythm status, were not available at the time of writing. Based on established surveillance protocols for adult congenital heart disease and valve-sparing aortic surgery, we recommend comprehensive follow-up comprising: (1) transthoracic echocardiography at 1, 3, 6, and 12 months postoperatively, then annually, with specific measurement of aortic root diameters at the annulus, sinuses of Valsalva, and sinotubular junction; (2) quantitative assessment of aortic regurgitation using vena contracta and regurgitant volume; (3) evaluation of right ventricular systolic pressure, pulmonary regurgitation fraction, and residual right ventricular outflow tract obstruction; (4) 24-hour Holter monitoring to assess rhythm status and atrial fibrillation burden; and (5) cardiopulmonary exercise testing to objectively quantify functional capacity. These data are essential to define late durability of the remodeled root in this unique population.

### Clinical significance

This case extends prior VSARR experience in congenital heart disease to the specific scenario of completely unoperated adult TOF, demonstrating that valve-sparing strategies can be integrated into primary repair even after decades of abnormal aortic flow exposure. The detailed technical description and perioperative management strategies provided here offer practical guidance for surgeons encountering similar patients.

## Conclusions

This case contributes to the limited literature on combining Yacoub valve-sparing aortic root replacement with primary intracardiac repair of tetralogy of Fallot in unoperated adults. The approach successfully preserved native aortic valve function, restored root geometry, and eliminated severe regurgitation while avoiding prosthetic valve-related morbidity and lifelong anticoagulation (3, 5).

Key implications include:

1. Expanded surgical armamentarium: VSARR should be considered in adult TOF patients with aortic root pathology, regardless of prior repair status (3, 5–7)
2. Technique individualization: The Yacoub remodeling technique is appropriate when sinuses are preserved and annular dilation is absent, whereas reimplantation may be preferred when annular stabilization is required (3)
3. Single-stage feasibility: Combined intracardiac and aortic procedures can be performed safely with appropriate perioperative management, including ASD fenestration and inflammatory modulation (8–13)
4. Anti-inflammatory strategies: Proactive inflammatory modulation may mitigate the risks of prolonged bypass in this population (8–13)

Long-term questions remain regarding durability of the remodeled root and preserved cusp function after decades of preoperative volume overload. Prospective multi-center registries with serial imaging and functional assessment are essential to define late survival, freedom from reoperation, and quality-of-life outcomes. Comparative studies should evaluate whether the reimplantation technique offers superior annular stability in this cohort (3). Emerging technologies, including personalized external root support and bioengineered leaflets, may further refine surgical options for adults with TOF-associated aortopathy.

## Data Availability

The original contributions presented in the study are included in the article/Supplementary Material, further inquiries can be directed to the corresponding author.
